# Sperm cellular and nuclear dynamics associated with ram fertility

**DOI:** 10.3389/fvets.2025.1577004

**Published:** 2025-05-19

**Authors:** Mustafa Bodu, Mustafa Hitit, Ayse Sari, Mesut Kirbas, Bulent Bulbul, Mehmet Bozkurt Ataman, Mustafa Numan Bucak, John Parrish, Abdullah Kaya, Erdogan Memili

**Affiliations:** ^1^Cooperative Agricultural Research Center, College of Agriculture, Food and Natural Resources, Prairie View A&M University, Prairie View, TX, United States; ^2^Department of Reproduction and Artificial Insemination, Faculty of Veterinary Medicine, Selcuk University, Konya, Türkiye; ^3^Department of Genetics, Faculty of Veterinary Medicine, Kastamonu University, Kastamonu, Türkiye; ^4^Department of Reproduction and Artificial Insemination, Faculty of Veterinary Medicine, Necmettin Erbakan University, Konya, Türkiye; ^5^Bahri Dagdas International Agricultural Research Institute, Konya, Türkiye; ^6^Department of Reproduction and Artificial Insemination, Faculty of Veterinary Medicine, Dokuz Eylul University, Izmir, Türkiye; ^7^Department of Animal and Dairy Sciences, University of Wisconsin-Madison, Madison, WI, United States

**Keywords:** ram, fertility, DNA integrity, sperm head, Fourier analysis

## Abstract

The aim of this study is to analyze DNA integrity, shape morphology, and membrane integrity in sperm from low-fertility (LF) and adequate- or normal-fertility (AF) rams. Various sperm evaluation methods such as sperm chromatin dispersion assay, Fourier harmonic amplitude (FHA) analysis, and other image analysis of morphometric parameters were used. An additional aim is to employ new statistical models with high reliability to predict ram fertility based on sperm head morphology parameters. Fresh semen was collected from 41 AF (conception rate 95.1% ± 0.6%) and 27 LF (conception rate 79.7% ± 2.5%) rams using artificial vagina. Differences (*p* < 0.05) were observed in percent motile sperm (mean ± SEM, 64% ± 3%, 72% ± 2%), percent viable sperm (78 ± 2%, 84 ± 1%), and head and acrosome abnormalities (1.9% ± 0.4%, 3.4% ± 0.4%) between LF and AF rams. The findings of different analyses also showed that the fertility of rams is not associated with DNA fragmentation (*p* > 0.05). Using the FHA analysis, an average head shape of ram sperm was constructed and harmonic amplitude 2 was determined, which tended to differ between the two ram fertility groups (*p* = 0.059). Based on the FHA and morphometric analysis, a significant linear discriminant model was constructed (*p* = 0.0013), which allowed for specificity in identifying LF rams (6/9, 66.7%) and sensitivity in identifying AF rams (39/47, 83.0%). The overall error rate remained good, which was 11/56 (20%). The findings of this study suggest that sperm DNA damage might not be used to predict ram fertility and that the statistical model based on the FHA analysis can be a potential tool in predicting ram fertility.

## 1 Introduction

It is estimated that the global population will exceed 9 billion in the second half of the 21^st^ century (1), which necessitates an increase in food production by more than 60% in the agriculture sector. Based on this trend, a substantial increase in the production of animal proteins is anticipated, including meat and milk. Consequently, the significance of the sheep farming industry will increase to fulfill the increasing demands (2, 3). Fertility refers to the capacity of fully developed male germ cells to fertilize the egg, sustain embryonic growth and development, and ultimately produce viable progeny (4). To ensure profitable, sustainable, and efficient livestock production, understanding fertility attributes is indispensable (5, 6). Therefore, in precision sheep farming, an accurate evaluation of sperm fertility potential is crucial for the evaluation of semen quality and male fertility.

Molecular health of sperm chromatin is critical for embryonic development and successful fertilization (7–9). Spermatogenesis involves the remodeling of nucleosomes in male germ cells, which may comprise complete or partial substitution of histones for protamines (10). At the final stages of spermatogenesis, sperm chromatin becomes extremely compacted and resistant to fragmentation (11). Even though sperm with an aberrant chromatin structure can successfully fertilize oocytes, the embryo that subsequently develops is vulnerable to damage. Studies have shown that deficiencies in the packaging or structure of chromatin are associated with infertility (12, 13). Accordingly, factors such as oxidative stress, apoptosis, and protamination failures lead to chromatin damage and sperm DNA fragmentation, either individually or in combination (14).

Conventional methods to evaluate sperm DNA include comet assay, sperm chromatin structure assay (SCSA), terminal transferase UTP nick-end labeling (TUNEL) assay, and sperm chromatin dispersion test (15–17). In addition, sperm head morphology has been identified as a biomarker for motility, fertility, and DNA fragmentation in bulls (18–20), stallions (21, 22), buffalo bulls (23), and goat (24). Using the Sperm-Class Analyzer (Microptic S.L., Barcelona, Spain), which analyzes motility, concentration, morphology, DNA fragmentation, and vitality, significant variations were observed among adult rams in sperm head morphological parameters, such as area, perimeter, length, and breadth (25). Furthermore, variations in ram fertility are reported to be significantly correlated with the proportions of spermatozoa with short and elongated heads in the ejaculate (26). Conventional semen analysis methods, such as manual microscopy to evaluate sperm motility and morphology, remain widely used but are limited by subjectivity and interobserver variability (27, 28). These techniques often fail to detect subtle defects in nuclear maturity and sperm structure (29, 30). In contrast, modern approaches such as computer-assisted sperm analysis (CASA), DNA fragmentation assays (e.g., TUNEL, comet, SCSA), and microfluidic sperm sorting provide objective, reproducible, and functionally informative evaluations (31–33). These technologies enable precise assessment of sperm quality, including chromatin integrity and oxidative damage, thus providing superior prognostic value for fertilization and pregnancy outcomes compared with traditional methods (34, 35). Martínez-Rodríguez et al. (36) have shown that sperm head morphology is a significant determinant in the ability of sperm to cross the mucus surrogate barrier and is associated with fertility. Compared with the sperm of other species, ram sperm contain a higher proportion of polyunsaturated fatty acids than cholesterol/phospholipids, making the sperm membrane susceptible to oxidative injury (37, 38). The functions of the sperm plasma membrane are critical for oviduct cell interactions, sustained metabolic processes, acrosome reaction, and capacitation. Thus, the loss of integrity of the sperm membrane reduces sperm viability (39, 40).

Fourier harmonic amplitude (FHA) analysis predicts the fertility of bulls (41) using computer-assisted image analysis that evaluates the shape of nuclei, which is expressed in harmonic amplitudes (HAs), to detect subtle shape differences in sperm nuclei. The parameters used to describe the curvature and perimeter of the sperm head are expressed as means. It is important to note that the FHA analysis does not distinguish between normal and aberrant conditions; instead, it provides the mean value associated with bull fertility for a specific male (42). This method has been developed based on the finding that the fertility rate of a population is negatively correlated with the intensity of DNA staining in the sperm head (43). Sperm DNA accounts for 90% of the sperm head, and sperm head morphology is determined by the arrangement and packaging of the DNA (44). Sperm head morphology corresponds to any alteration in the configuration and packaging of the chromatin within the sperm cell (45, 46). The FHA analysis uses HAs, which are multivariate shape measurements, to account for the curvature of the sperm perimeter (19, 42), and HAs are correlated with damaged DNA in the sperm chromatin structure assay (7).

Even though numerous studies have focused on sperm nuclear dynamics (25, 26, 36, 47), significant knowledge gaps still exist regarding sperm chromatin integrity and sperm head shape morphology in the context of ram fertility. To achieve a better understanding of the cellular and nuclear dynamics of ram sperm, comparative analyses of sperm from AF and LF ram are required. The present study aims to determine which of the sperm nuclear and cellular dynamics parameters are related to ram fertility. The findings provide novel insights into the assessment of sperm quality and prediction of ram fertility.

## 2 Materials and methods

### 2.1 Determination of ram fertility

The Ministry of Agriculture Animal Care Committee approved the experiments carried out in this study (no: 22.12.2016/58). Merino rams from the Bahri Dagdas International Agricultural Research Institute in Konya, Türkiye, were used. Each animal was kept under good conditions following a similar protocol. Rams with natural mating records from the 2017–2019 breeding seasons were selected to determine the ram fertility phenotype, which was defined based on pregnancy outcomes confirmed by both non-return rate (NRR) percentage and ultrasonographic examination 35 days post-mating. Fertility scores of rams were calculated using teaser rams during the initial heat detection of the females. The ewes that were in heat were kept in separate breeding pens. Then, one ram was introduced into the pen to achieve natural mating. The ewes and rams were separated following the confirmation of a successful mating. The same procedure was replicated throughout the regular breeding season (from early September to late November) using several ewes for each ram. A teaser ram was used to determine whether the ewes were returning to the estrous cycle from day 12 to day 25 upon mating and returned to the same flock. In addition, 35 days after insemination, pregnancy was confirmed using an ultrasonographic examination with a 6/8 MHz transrectal linear array transducer (Pie Medical 480, 100 LC, Holland). The fertility score was calculated based on each ram's NRR percentage and pregnancy confirmation by ultrasonographic examination. Throughout the lambing season, lambing rates were recorded for each ram.

Rams were classified into two fertility groups based on their conception rates from natural matings recorded between 2017 and 2019 (Table 1). Rams with a conception rate of 1 SD below the overall population mean were classified as the low-fertility (LF) group (*n* = 41, conception rates 95.1% ± 0.6%), and the remainder were classified as the adequate-fertility (AF) group (*n* = 27, conception rates 79.7% ± 2.5%). The mean conception rate of the overall population was 89.0% ± 0.8%. Exclusion criteria included missing records, single mating events, and signs of reproductive abnormalities.

**Table 1:** Pregnancy rates of rams used for insemination during the breeding seasons in 2017–2019.

**Groups**	***n***	**Conception rate (%)**
Adequate fertility	41	95.1 ± 0.6^*^
Low fertility	27	79.7 ± 2.5
Overall fertility	68	89.0 ± 0.8

Mean ± SEM pregnancy rates from different ram fertility groups. ^*^denotes the difference between adequate- and low-fertility groups (*p* < 0.05).

### 2.2 Semen collection

Semen was collected from the AF and LF Merino rams using an artificial vagina as described by Salomon (48). Fresh semen was immediately kept in a water bath (37°C) in a graduated test tube, and its volume was noted. Semen was visually examined for potential contaminants such as feces, urine, blood, debris, abnormal colors, or odors; however, since no such contamination was observed in any of the samples, all ejaculates were considered suitable for further evaluation. Ejaculate volume, sperm concentration, percentage of motile spermatozoa, and wave motion were evaluated immediately after collection (49).

### 2.3 Determination of semen volume

After the collected semen was carefully transferred to the graduated test tube, its volume was accurately measured using the graduation marks.

### 2.4 Determination of semen quality

#### 2.4.1 Mass activity

Sperm mass activity was determined following the method of Evans and Maxwell (48). To assess wave motion using a phase-contrast microscope (400x magnification), 5 μl of raw semen was dropped onto a pre-warmed glass slide (37°C), which was scored between 0 and 5 (0 no movement; 1 very slow movement with no swirl; 2 slow movement with weak swirl; 3 good movement with good swirl; 4 fast movement with good swirl; and 5 fast movement and swirl) (50).

#### 2.4.2 Sperm concentration

Sperm concentration was determined using a hemocytometer as described by Evans and Maxwell (48). In this method, 5 μL of raw semen was transferred to a 1-ml tube and diluted with 995 μL of Hayem's solution (5 g Na_2_SO_4_, 0.5 g HgCl_2_, 1 g NaCl, and 200 mL double-distilled water). Following mixing, sperm suspensions were counted using a Thoma counting chamber (Paul Marienfeld GmbH & Co. KG, Lauda-Königshofen, Germany) after three replicates of each sample at 400x magnification.

#### 2.4.3 Motility

Sperm motility was evaluated using phase-contrast light microscopy, following the methodology outlined by Evans and Maxwell (48). Raw semen was diluted to 100 × 10^6^ cells/ml with phosphate buffer saline (PBS) (P4417, Millipore Sigma, USA). Then, 10 μL of semen samples was dropped onto a pre-warmed (37°C) microscope slide and mounted with a coverslip (22 × 22 mm). For each semen sample, sperm motility was estimated subjectively in five microscopic fields under a phase-contrast microscope (400 × ), and the average of the five different fields was recorded as the final motility rate (48).

### 2.5 Sperm morphology evaluation

Morphological abnormalities were investigated as described by Kaya et al. (51) using phase-contrast microscopy (1,000 × magnification) after 300 spermatozoa cells from the samples were fixed in Hancock's solution, which was prepared by mixing 62.5 ml formalin (37%), 150 ml PBS, and 150 ml physiological saline (0.9% NaCl). Approximately 15 μl of semen was taken in a 1.5-mL tube containing 1 mM Hancock's solution. At least 10 μl of the sperm suspension was dropped onto a slide and mounted with a coverslip. The types of morphological anomalies recorded included detached head, alterations of the head (decapitated and macro- and microcephaly), presence of cytoplasmic droplets, tail defects (coiled, bent, or broken tails), and midpiece abnormalities (double midpiece), as described in a previous study (51).

### 2.6 Sperm membrane integrity

Sperm membrane integrity or viability was determined using eosin–nigrosine staining as previously described by Moradi et al. (49). In brief, 5 μl of the semen sample diluted with 10 μl of eosin–nigrosin stain (nigrosin 242.48 mM, eosin-Y 25.77 mM, sodium citrate 112.37 mM) was placed on a pre-warmed slide. Sperm with an unstained head were considered membrane intact (viable), and those with a red or dark pink head were considered membrane-damaged (dead). The percentage of viable sperm was analyzed by counting at least 200 spermatozoa per slide under a phase-contrast microscope (400 × ).

### 2.7 Sperm DNA integrity assessment (Halomax)

Sperm DNA fragmentation was evaluated using Halomax kits (Halotech DNA, Madrid, Spain) from Halotech DNA (Spain) according to the manufacturer's instructions (16). In this method, a large halo is associated with DNA fragmentation because of the absence of a denaturing treatment, and it is assumed that the extraction of nuclear proteins from the spermatozoa containing fragmented DNA releases DNA fragments between two strand breaks. Finally, sperm nuclei disperse chromatin, forming a low-stained peripheral halo that is distinguishable under low magnification. Briefly, sperm concentration was adjusted to 25 × 10^6^ cells/mL by diluting with PBS. Then, 25 μL of the sperm suspension was pipetted into a tube containing 50 μL of low-melting agarose liquid at 37°C, and the mixture was placed in a well of a pretreated slide provided in the kit and mounted with a coverslip (24 × 24 mm). The covered slide was incubated at 4°C for 5 min. The coverslips were gently removed, and the slide was immediately transferred to a lysis solution (supplied with the kit) at room temperature for 5 min. Then, the slides were rinsed with distilled water and treated with graded ethanol series (70%, 90%, and 100% ethanol) for 2 min. Following dehydration, the slides were stained with propidium iodide, and at least 300 spermatozoa per slide were examined under a fluorescent microscope (Leica DM3000, Germany) with a 40 × objective. The results of the chromatin dispersion test were investigated according to the manufacturer's instructions.

### 2.8 Sperm nuclear shape analysis

#### 2.8.1 Labeling nuclear DNA with Hoechst 33342

Following collection, 100 μL of raw semen was pipetted into a 1.5-mL vial containing 400 μL of 2.9% sodium citrate solution. Then, 2.5 μL of Hoechst 33342 solution (stock solution 5 mg/mL) was added to the sperm suspension, and the mixture was incubated for 30 min at 37°C. Following incubation, 250 μL of 2.9% sodium citrate solution was added and centrifuged at 6,000 *g* for 3 min at room temperature. The supernatant was aspirated, and 650 μL of a fixative solution (0.4% paraformaldehyde in 2.9% sodium citrate solution) was added to the tube and incubated at room temperature for 5 min. The suspension was centrifuged at 6,000 *g* for 15 s as the fixed sperm sedimented faster. The supernatant was discarded, and 700 μL of 2.9% sodium citrate solution was added to the tube. The suspension was again centrifuged at 6,000 *g* for 15 s. The supernatant was then aspirated, and the pellet was resuspended with double-distilled water. This last step was repeated twice to remove all citrate solution. Finally, the last pellet was resuspended with 500 μL double-distilled water, and then 10 μL of sperm suspension was placed onto a slide and spread gently. The slides were dried under a stream of air from a blow drier on a slide warmer at 37°C. The dried slides were stored in slide boxes and kept in a desiccator at room temperature until covering.

#### 2.8.2 Phase-contrast and epifluorescence imaging

In this method, 3.5 μL of 0.22 mM triethylenediamine (Sigma-Aldrich (Merck KGaA), Darmstadt, Germany) (DABCO) was mixed with one part of PBS and nine parts of glycerol on top of the dried sample on the slide to prevent florescent fading while obtaining images. The slides were mounted with an 18 × 18 mm coverslip, and clear nail polish was used to seal the edges. The slides were imaged within 24 h following coverslip application. Images were obtained using a Nikon Microphot-FX microscope (Nikon Corporation, Tokyo, Japan) configured for phase contrast microscopy, epifluorescence, and computer image analysis. An OSRAM 100-watt HBO Mercury Short Arc lamp (OSRAM GmbH, Munich, Germany) was used as the source of fluorescence that transmitted light through filters. Two images were taken for each field: a phase-contrast image and an epifluorescent image of Hoeschst-33342-stained sperm nuclei, using a filter cube with a 400-nm dichromatic mirror, a barrier filter of >400 nm emission, and a 365 ± 20 nm excitation filter. Images were obtained using a Nikon Fluor 40X Ph3DL (Nikon Corporation, Tokyo, Japan) 0.85 numerical aperture objective lens and a 1.25 magnifier and captured by a QIClick Camera (QImaging (Teledyne Photometrics), Surrey, BC, Canada) (mono 12-bit 01QIClick-F-M-12 model) at an exposure of 125 ms. Approximately 130 sperm were imaged per sample. Then, 100 sperm without visibly interrupted perimeters were randomly selected per sample that were counted during imaging.

#### 2.8.3 Image analysis and data collection

**Figure 1:**
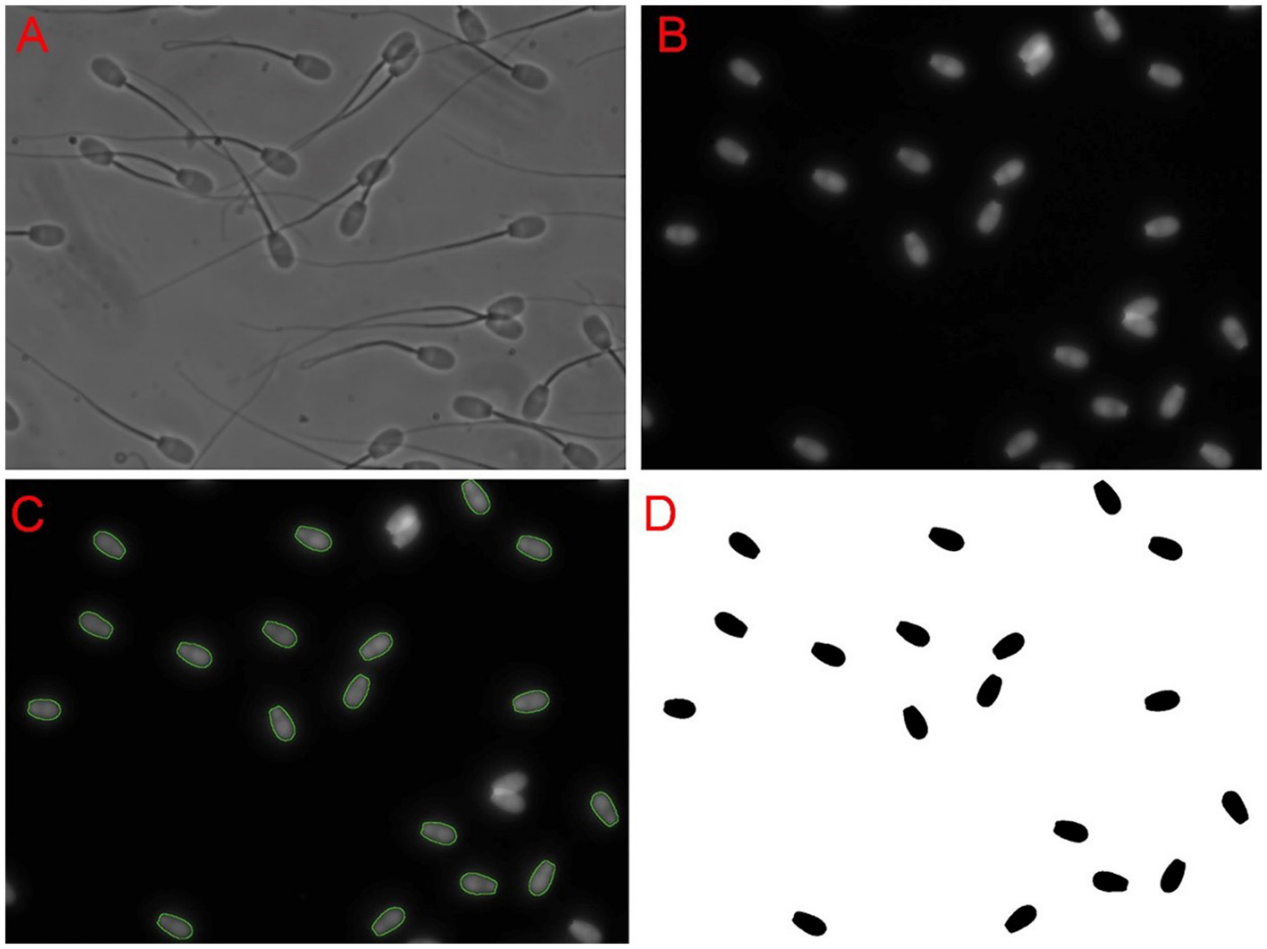
Sperm nuclei selection approach. First, a phase-contrast image **(A)** was obtained. Then, an epifluorescent image **(B)** of Hoechst nuclei staining on the same field was captured. The software used identified the perimeter outlines of the sperm nuclei, which are is shown as a green overlay on the Hoechst image **(C)**. Overlapping sperm nuclei and incorrectly identified objects were deleted. Selected spermatozoa nuclei were filled in and once again checked to ensure they were indeed correctly identified **(D)**. It was these objects that were then used in image analysis.

**Figure 2:**
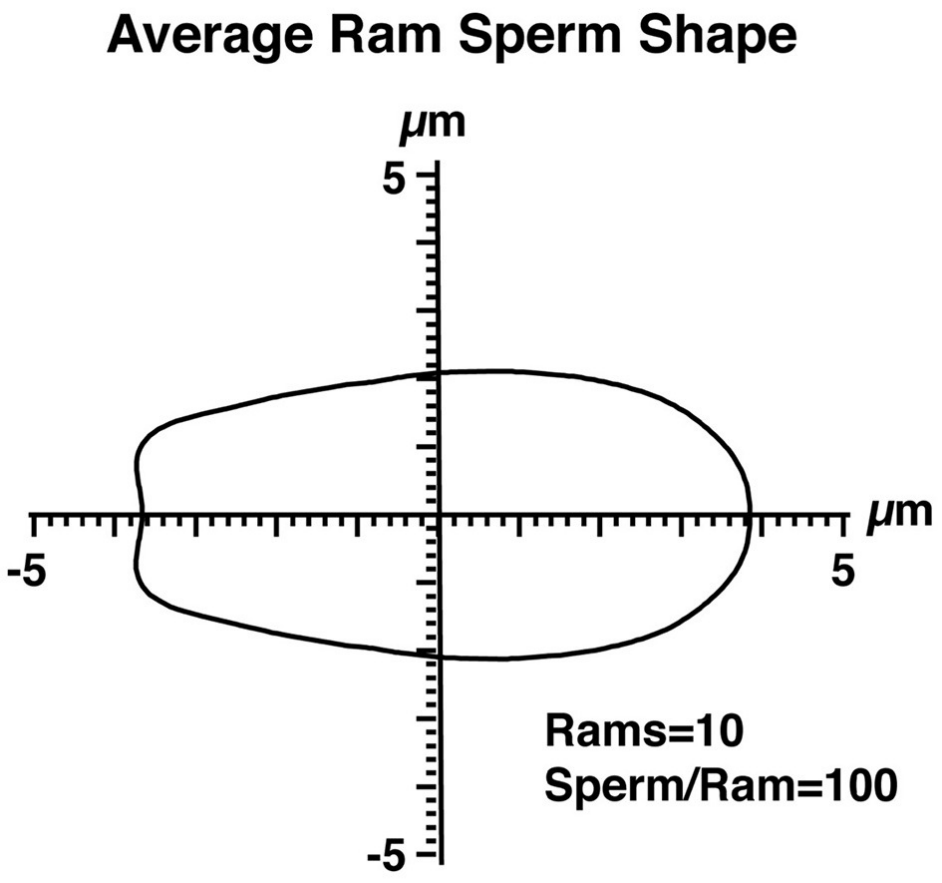
The average shape of ram sperm nuclei. The perimeter coordinates of 1,000 ram sperm, obtained from 10 rams (100 sperm per ram), are shown in Cartesian coordinates. The axes intersect at the center of mass of the sperm nuclei.

The assessment of sperm nuclear shape via FHA analysis and other morphometric measures were conducted using the ImageJ 1.51w image analysis software (Wayne Rasband, National Institute of Health) along with custom macros and plugins for sperm analysis developed by Dr. Parrish's laboratory (Department of Animal and Dairy Sciences, University of Wisconsin-Madison, USA), which are available upon request. The general approach to identifying sperm nuclei is shown in Figure 1. Briefly, phase-contrast and epifluorescent images were taken from the same unit of area. The imaging software then identified and outlined sperm heads using a Laplacian thresholding approach and provided an opportunity to remove non-sperm objects or sperm with interrupted perimeters. The perimeters of sperm nuclei from the Hoechst image were then overlayed on the phase-contrast image to ensure that the perimeters identified sperm nuclei correctly. Any sperm nuclei with incorrect boundaries were deleted from the analysis. The perimeter data were the output for further analysis using the Statistical Analysis System (SAS Institute, version 9.4). To begin the SAS analysis, Cartesian coordinates defining the nuclear perimeters of 100 randomly selected sperm nuclei per ram were obtained. These coordinates were transformed into polar coordinates and Fourier series using trigonometric regression. The mean Fourier functions were determined, and the mean HAs 0 to 5 (HA0–HA5) were sufficient to depict multivariate measures of sperm nuclear shape, as described in a previous study (19). The impact of HA0–HA5 on sperm nuclear shape was described (19). Briefly, HA0 affects the overall sperm size; HA1 affects the curvature of the nuclear anterior region; HA2 is involved in sperm head elongation; and HA3, HA4, and HA5 represent tapering of the posterior head region. The average nuclear shape of ram sperm was determined by averaging the perimeter coordinates of 1,000 ram sperm, 10 rams, and 100 sperm/ram (Figure 2). The dispersion of HAs was also determined to include variance, skewness, and kurtosis, which were then quantified in SAS. ImageJ provided the ability to generate morphometric data on a pixel-by-pixel approach from the identified objects, which were, in this case, sperm nuclei and included area, mean gray value (intensity), standard deviation of intensity, perimeter, circularity, aspect ratio, roundness, solidity, median intensity, skewness of intensity, and kurtosis of intensity (definitions can be found within ImageJ User Guide; https://imagej.net/ij/docs/guide/index.html).

### 2.9 Statistical analysis

Semen parameters, including mass activity, percentage of motile sperm, concentration, percentage of abnormal sperm, viable sperm percentage, sperm DNA fragmentation rate, and fertility rate, were compared between AF and LF rams using an independent-sample *T*-test (IBM SPSS Statistics 22.0) (52). The data were presented as mean ± standard error (SE). *P* values < 0.05 were deemed statistically significant. To correctly predict the fertility rate based on nuclear shape and morphometric parameters, a total of 56 rams were evaluated (*n* = 9 and *n* = 47 in the LF and AF groups, respectively) using linear discriminant analysis of variance within SAS. The linear discriminant analysis compared the two groups for univariate differences in individual variables, canonical correlation, the square of the canonical correlation for multivariate analysis of variance using maximum likelihood evaluation, and predictive model cross-validation to calculate percent true positive (sensitivity) and percent true negative (specificity) along with the overall error rate (false positive + false negative/total sample size) (53). The cross-validation approach provides a simulation of how the model would perform in practice on new observations if a second dataset is not available, as was in our case.

## 3 Results

### 3.1 Fertility differences between rams

Fresh semen was collected from a total of 68 rams using an artificial vagina in 2017 and 2019. The mean population fertility score of the rams was 89.0 ± 6.6, which represents the percentage of ewes that were confirmed pregnant from a single mating. Rams with a conception rate that was 1 SD below the overall population mean were categorized as the LF group (*n* = 27; 89.0–6.6 = 82.4), with a mean ± SEM of 79.7% ± 2.5%. The remainder were categorized as the AF group (*n* = 41; >82.4) with a mean ± SEM of 95.1% ± 0.6% (Table 1). Different subsets of rams from the AF and LF groups were available for individual experiments as some of the 2017 semen samples remained. The information on the number of rams used in individual experiments is described in the following sections.

### 3.2 Sperm membrane integrity and morphology

Sperm mass activity, motility, concentration and viability (Table 2), and sperm abnormalities (Table 3) were determined for 38 AF and 16 LF rams. These rams were available for the analysis of membrane integrity and morphology. The percentage of motile sperm and the percentage of viable sperm were higher in AF rams than in LF rams (*p* < 0.05), but there was no effect of ram fertility on mass activity or concentration (*p* > 0.05). In addition, there was no effect of ram fertility on the percentage of normal, midpiece, tail, or cytoplasmic droplets (*p* > 0.05), but a slight increase in head and acrosome abnormalities was observed in the AF group (*p* < 0.05).

**Table 2:** Assessment of sperm mass activity, motility, concentration, and viability parameters of high- and low-fertility rams.

**Fertility groups**	***n***	**Mass activity**	**Motility (%)**	**Concentration (10^9^/mL)**	**Viable (%)**
Adequate	38	3.1 ± 0.2	72 ± 2^*^	2.02 ± 0.20	84 ± 1^*^
Low	16	2.9 ± 0.2	64 ± 3	1.99 ± 0.47	78 ± 2

Mean ± SEM sperm parameters from different ram fertility groups. ^*^ denotes the difference between adequate- and low-fertility groups (*p* < 0.05).

**Table 3:** Percentages of sperm abnormalities from high- and low-fertility rams.

**Fertility group**	***n***	**Normal (%)**	**Head and acrosome (%)**	**Mid-piece (%)**	**Tail (%)**	**Cytoplasmic droplets (%)**
Adequate	38	88.4 ± 0.9	3.4 ± 0.4^*^	2.1 ± 0.3	4.8 ± 0.5	1.3 ± 0.2
Low	16	89.3 ± 1.4	1.9 ± 0.4	1.4 ± 0.5	6.3 ± 1.2	1.2 ± 0.4

Mean ± SEM sperm abnormalities from different ram fertility groups. ^*^ denotes the difference between adequate- and low-fertility groups (*p* < 0.05).

### 3.3 Sperm chromatin dispersion assay

**Figure 3:**
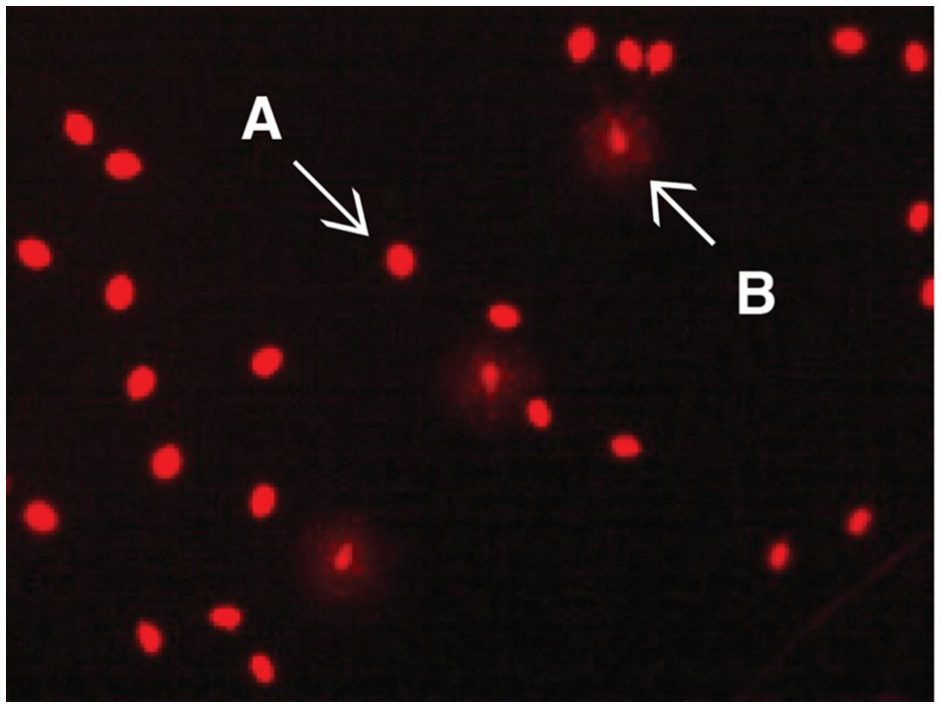
Evaluation of the DNA damage rate in sperm using Halomax/Halotech DNA method. Bright red halo-free spermatozoa are those without DNA damage **(A)**; pale red spermatozoa with halo around them are those with DNA damage **(B)**. No significant difference was observed in adequate and low fertility rams (*p* > 0.05).

Findings regarding sperm DNA fragmentation for 18 AF and 13 LF rams are presented in Table 4. The decrease in the number of rams was due to limited kits for the sperm chromatin dispersion assay. Sperm with large nucleoids and a spotty halo of dispersed chromatin were considered sperm with fragmented DNA (Figure 3), whereas those with small nucleoids and a compact halo of chromatin were considered sperm without fragmented DNA. There was no effect of ram fertility on the percentage of sperm with fragmented DNA (*p* > 0.05).

**Table 4:** Sperm DNA fragmentation (%) evaluated from high- and low-fertility rams.

**Fertility group**	***n***	**DNA damage (%)**
Adequate	18	5.0 ± 0.6
Low	13	3.6 ± 0.8

Mean ± SEM sperm abnormalities from different ram fertility groups. There was no difference between adequate- and low-fertility groups (*p* > 0.05).

### 3.4 Fourier harmonic amplitude and morphometric analysis

The FHA analysis provides an objective approach to describing the shape of sperm nuclei or sperm head. The details on the average shape of ram sperm for HA0–5 are presented in Table 5 based on ram fertility. A trend for differences was observed only in HA2 (*p* = 0.059), while other HAs did not differ between the two fertility groups (*p* > 0.05). To determine whether HAs can predict ram fertility, linear discriminant analysis was used, which showed no differences between the two fertility groups (*p* = 0.208, canonical correlation = 0.391, canonical correlation squared = 0.153); thus, canonical correlation was not different from 0 and the model was not related to fertility groups. Therefore, a linear discriminant analysis was conducted using all 38 variables from FHA mean values and dispersion values, and morphometric analysis was carried out using a stepwise approach to reduce the large number of variables. After six rounds of iterations, a model containing roundness, circularity, standard deviation of intensity, variance of HA5, variance of HA4, and skewness of HA4 was found, but only roundness differed between the two fertility groups (*p* = 0.048). The model's canonical correlation was 0.747, which was significant (*p* < 0.0001). However, when the predictive ability of the model was evaluated using cross-validation to determine how the model might act when provided with real data, it identified 43/47 (91% specificity) in the AF group correctly but correctly predicted only 3/9 (33% sensitivity) in the LF group. The overall error rate was 10/56 (18%). Therefore, sensitivity to identify LF rams was low, which is the most important feature. Numerous prediction models were then evaluated using linear discriminant analysis before selecting the model presented in Table 6, which was the best model with the fewest parameters. The cross-validation presented in Table 7 shows that the final model had both good specificity at identifying LF rams (6/9, 66.9%) and sensitivity at identifying AF rams (39/47, 83.0%). The overall error rate remained good at 11/56 (20%).

**Table 5:** Harmonic amplitude (HA0–5) parameters used to define sperm head shape in rams by fertility groups.

**Harmonic amplitude**	**Mean** ±**SEM**		
	**AF (*n =* 47)**	**LF (*n =* 9)**	**Overall (*n =* 56)**
HA0	2.851 ± 0.010	2.838 ± 0.029	2.849 ± 0.009
HA1	0.085 ± 0.002	0.087 ± 0.005	0.085 ± 0.002
HA2	0.905 ± 0.006^†^	0.872 ± 0.024	0.900 ± 0.006
HA3	0.078 ± 0.002	0.082 ± 0.006	0.079 ± 0.002
HA4	0.153 ± 0.003	0.143 ± 0.011	0.151 ± 0.003
HA5	0.024 ± 0.001	0.024 ± 0.002	0.024 ± 0.001

Adequate-fertility (AF) and low-fertility (LF) groups. ^†^Trend for a difference from LF, *p* = 0.059.

**Table 6:** Relationship with sperm shape and morphometric analysis parameters by ram fertility groups^a^.

**Parameters^b^**	**Mean** ±**standard error**^**c**^	
	**Adequate (*n =* 47)**	**Low^d^ (*n =* 9)**
Mean HA2 (μm)	0.905 ± 0.006	0.872 ± 0.023^†^
Mean intensity (gray-level value)	99.168 ± 3.015	110.737 ± 10.719
Standard deviation of intensity	17.754 ± 0.607	20.528 ± 2.495
Perimeter (μm)	21.415 ± 0.075	21.245 ± 0.287
Area (μm^2^)	28.257 ± 0.185	27.931 ± 0.586

^a^Adequate-fertility (AF) and low-fertility (LF) groups. ^b^These were the parameters included in the linear discriminant analysis. ^c^Standard error values represent the variation between rams within a fertility group. ^d^Based on multivariate analysis (MANOVA) with the Wilks lambda test, the LF group was different (*p* = 0.0013). ^†^The values in the same line tended to differ with a univariate F test (*p* = 0.059).

**Table 7:** Cross-validation summary for fertility groups using linear discriminant functions^a^.

**Fertility groups**	**Cross-validation prediction rates**	
Adequate	True negative 83.0% (39/47)	False negative 17.0% (8/47)
Low	False positive 33.3% (3/8)	True positive 66.7% (6/9)

^a^The model used was that shown for parameters in Table 6. Canonical correlation ± SE was 0.57 ± 0.09 (*p* < 0.001; test correlation not equal to 0, using maximum likelihood ratio). Total number of rams = 56. Rams were predicted to be in the correct fertility group with good specificity at identifying low-fertility rams (6/9, 66.7%) and good sensitivity to identify adequate-fertility rams (39/47, 83.0%) with an overall error rate of only 11/56 or 20%.

## 4 Discussion

Morphological abnormalities of sperm and defects of nuclear components are indicators of sperm fertilizing ability. In this study, we analyzed sperm cellular and nuclear dynamics associated with ram fertility by evaluating sperm morphology and membrane integrity using sperm chromatin dispersion (Halomax) assessment and computer-assisted fluorescent staining of nuclear shape, as well as conventional methods for sperm evaluation.

In this study, no differences in DNA fragmentation were observed between sperm from the AF and LF groups based on DNA integrity assessment. However, LF rams tended to show a higher relative DNA fragmentation abundance than AF rams (*p* = 0.170). In addition, HA2 (indicator of nuclear length) tended to be lower in LF ram sperm (*p* = 0.059), but there was no significant difference in HA0, HA1, HA3, HA4, and HA5 between the two groups (*p* > 0.05). Furthermore, a new reliable statistical model was developed using linear discriminant analysis to predict ram fertility using HA and morphometric parameters of mean HA2, mean intensity, standard deviation of intensity, perimeter, and area.

Relationships between DNA fragmentation, abnormal sperm morphology, chromatin structure, and FHA in bulls have been reported in several studies (16, 44, 54). In the past few decades, there has been an increasing body of research investigating the role of sperm DNA integrity in male infertility (55). Evidence is accumulating that DNA damage is higher in the spermatozoa of infertile males compared to fertile males (56–58). In dairy bulls, DNA damage accounted for significant variations in fertility, and the proportion of spermatozoa with DNA damage was more than 2-fold higher in bulls with below-average fertility than in those with above-average fertility (59).

Boe-Hansen et al. (60) also confirmed the role of sperm DNA integrity in fertility and suggested that the presence of immature spermatogenic cells, cytoplasmic proximal droplets, and alterations in sperm head shape are associated with sperm DNA integrity and protamine deficiency. Similar reports on the role of sperm DNA integrity in fertility and/or semen quality are available for other farm animals, including stallions (61, 62), boars (63, 64), and rams (65, 66). However, abnormal chromatin structure and DNA fragmentation can also be observed in normal sperm and can be assessed using conventional sperm evaluation methods (54, 67–69). Therefore, DNA damage may not impair the fertilization ability of spermatozoa. A previous study showed that the DNA fragmentation rate measured using the sperm chromatin dispersion test is not correlated with the conception outcome of intrauterine insemination in humans (70). However, the rate of a fertilized oocyte and the resultant embryo depend on the degree of sperm DNA damage.

Sperm DNA damage occurs as a result of a wide range of events, including DNA–protein cross-linkage, base deletion or modification, interstrand or intrastrand cross-linkage, and single- or double-strand DNA breaks (71). Often, minor fragmentation in sperm chromatin can be repaired by the oocyte or even by the resultant embryo (72). As little as one double-strand break in DNA can be quite dangerous as it leads to the death of the cell by preventing the transcription of an essential gene or triggering apoptosis. Depending on the proportion of DNA damage in the sperm, different scenarios may arise after fertilization. For example, the fertilization of sperm with fragmented DNA into the egg may initiate embryonic development, but this may result in early embryonic death or abortion (73). In addition, even sperm with a high degree of DNA damage can fertilize the egg and support the developing embryo and fetus to term. In this scenario, paternal damaged DNA can affect offspring and give rise to congenital diseases, cancer, and even infertility (74–76). In the present study, no statistical difference in DNA damage was observed between sperm from LF and AF rams, but the tendency to DNA damage was higher in LF rams. As mentioned earlier, the degree of sperm DNA fragmentation can be an indicator of ram fertility if it is evaluated together with other fertility parameters. However, paternal DNA damage could be associated with the health of offspring rather than with ram fertility prediction. The integration of morphometric data and FHA analysis offers a novel framework for the objective quantitative assessment of ram fertility. This approach has the potential to be standardized and implemented in routine andrological evaluations, particularly for the early screening of sub-fertile males. Furthermore, its adaptability to high-throughput automated image analysis platforms makes it a promising candidate for cross-species application in advanced reproductive biotechnology programs.

Since the nucleus occupies a major part of the sperm head in mammals, any alterations in the nuclear component, including DNA and chromatin positions, may affect sperm head shape (54, 77, 78). In the FHA analysis, sperm nuclear shape was used to classify rams into AF and LF rams. It determines the DNA of sperm cells, and sperm DNA accounts for 90% of the sperm head (44). Previous studies have used FHA analysis to differentiate bulls (19, 42), boars (79), and water buffalo (80) based on fertility. Across multiple studies on bulls, different shapes of nuclear components were found to be related to fertility, including HA0, HA1, HA2, HA4, and HA5, as well as variation with individual HAs. In boars, HA2 and HA4 were found to be decreased in LA males. In general, in boars and bulls, LF sperm are slightly longer and more tapered (42), with normal or fragmented DNA. Fragmented DNA prevents sperm from fertilizing the egg, and even if fertilization occurs, it cannot support the development of an embryo to full term (19, 81) due to reasons yet to be resolved. Furthermore, the failure of motile sperm to sustain fertilization and pregnancy leads to decreased fertility in bulls (81).

In the present study, differences in sperm nuclear shape were observed at HA2 between AF and LF rams. Therefore, sperm from AF rams were more elongated and tapered than those from LF rams, which is consistent with previous studies (26, 82–84). A study carried out on humans reported that sperm head dimension influences the progressive velocity and amplitude of lateral head movement of sperm (85). In another study (84), higher-fertility male red deer showed higher percentages of fast and linear sperm with elongated and smaller heads. Furthermore, a previous study has shown that the proportion of sperm with elongated heads is associated with ram fertility (26). Sperm with elongated heads can be hydrodynamically more effective, which can influence their fertilization ability. In addition, because they can have less resistance to forward progression, they may be faster (84, 86). Furthermore, they may have a longer lifespan in the female reproductive tract because they may expend less energy (87). Moreover, some researchers have reported evidence supporting the participation of protamines in sperm head shaping, thus giving rise to smaller and longer sperm heads (88, 89).

This study has some limitations that should be acknowledged, which may guide future research. Although the sample size was statistically sufficient, expanding the number of animals and including different breeds and environmental conditions in future studies would enhance the generalizability of the findings. In addition, although the evaluation included advanced image-based techniques, only a single ejaculate per ram was assessed. Incorporating multiple ejaculates over time could provide a more comprehensive understanding of individual variability. Finally, the addition of functional assays such as *in vitro* fertilization and embryo development assessments would further validate the biological relevance of DNA fragmentation and morphometric parameters.

Other than the sperm chromatin dispersion test, several methods exist to evaluate sperm DNA damage, including SCSA, TUNEL, and comet assay. Results from different methods should neither be compared nor verified with each other (90). This indicates that the results from different methods are not interchangeable as the principal mechanism of each method is different (90). The sperm chromatin dispersion test quantifies the susceptibility of DNA to denaturation following acid denaturation and removal of nuclear proteins (91). Logistic regression analysis indicates that the sperm chromatin dispersion test can be applied to discriminate between men with normal and abnormal proportions of sperm DNA defects, with up to 70% accuracy (90). Numerous techniques are available to analyze sperm head. Of these, FHA analysis is an objective approach that describes multiple perimeter points of sperm nuclei shape, which is used in determining sperm chromatin structure. The success of the approach relies on novel uses of computer-aided image analysis, inclusion of sophisticated mathematics to evaluate sperm head shape, and statistical methods not commonly used in andrological studies. These evaluations may overturn previous concepts that associate sperm morphology with fertility (19, 83). However, no single application still appears to be reliable enough to determine clinically significant DNA fragmentation with high accuracy that predicts male fertility (90).

This study addresses critical knowledge gaps related to chromatin integrity and head shape morphology in the sperm of rams. Although previous studies have demonstrated associations between these parameters and fertility in other species, limited data are available on rams. By integrating chromatin dispersion testing, advanced morphometric measurements, and FHA analysis, this study provides a comprehensive evaluation of both nuclear integrity and sperm head structure in relation to fertility. Furthermore, the use of a novel statistical classification model further strengthens the potential practical application of our findings in routine fertility screening programs.

## 5 Conclusions

In conclusion, there were no differences in DNA fragmentation between sperm from LF and AF rams based on the sperm chromatin dispersion test. The findings of this study suggest that paternal fragmented DNA can be related to the health of offspring rather than to ram fertility prediction. Sperm nuclear shape, as assessed using the FHA analysis, can objectively be used for predicting ram fertility. Values of HA2 (indicator of nuclear length) tended to be lower in the sperm of LF rams, and thus, sperm of AF rams were more elongated. Sperm DNA damage might not be used to predict ram fertility, but the statistical model based on the FHA and morphometric analysis has the potential for predicting ram fertility.

## Data Availability

The datasets presented in this article are available upon request.
